# CD8^+^ T-cell responses to *Theileria parva* are preferentially directed to a single dominant antigen: Implications for parasite strain-specific immunity

**DOI:** 10.1002/eji.200939227

**Published:** 2009-09

**Authors:** Niall D MacHugh, Timothy Connelley, Simon P Graham, Roger Pelle, Principia Formisano, Evans L Taracha, Shirley A Ellis, Declan J McKeever, Alison Burrells, W Ivan Morrison

**Affiliations:** 1 The Roslin Institute, Royal (Dick) School of Veterinary Studies, University of EdinburghUK; 2 International Livestock Research InstituteNairobi, Kenya; 3 Institute for Animal HealthCompton, Berkshire, UK

**Keywords:** CD8^+^ T-cell, Immunodominance, Strain specificity, *Theileria parva*

## Abstract

Although immunodominance of CD8^+^ T-cell responses is a well-recognised feature of viral infections, its role in responses to more antigenically complex pathogens is less clear. In previous studies we have observed that CD8^+^ T-cell responses to *Theileria parva* exhibit different patterns of parasite strain specificity in cattle of different MHC genotypes. In the current study, we demonstrated that animals homozygous for the A10 and A18 MHC haplotypes have detectable responses to only one of 5 *T. parva* antigens. Over 60% of the responding T cells from the A18^+^ and A10^+^ animals recognised defined epitopes in the Tp1 and Tp2 antigens, respectively. Comparison of T-cell receptor β chain expression profiles of CD8^+^ T-cell lines and CD8^+^ T cells harvested *ex vivo* confirmed that the composition of the T-cell lines was representative of the *in vivo* memory CD8^+^ T-cell populations. Analysis of the Tp1 and Tp2 antigens revealed sequence polymorphism, which was reflected by differential recognition by T-cell lines. In conclusion, we have demonstrated a profound immunodominance in the CD8^+^ T-cell response to *T. parva*, which we propose is a major determinant of the parasite strain specificity of the response and hence immune protection.

## Introduction

CD8^+^ T-cell responses directed against class I MHC-bound antigenic peptides play a key role in immunity against many intracellular pathogens, including viruses, bacteria and parasites 1. Processing of proteins from such infectious agents can generate a large number of distinct peptides capable of associating with class I molecules 2, 3. Although the T-cell repertoire does not contain T cells specific for all of these peptides, multiple T-cell specificities potentially capable of responding to a range of peptides are usually present, even in the case of the simplest RNA virus 4. However, a characteristic feature of CD8^+^ T-cell responses to many viruses is that the peptide epitopes display a strong dominance hierarchy, resulting in focusing of the response on a limited number of the most dominant epitopes 3, 5, 6. In extreme cases, such as during infection with HIV-1 virus in humans, most of the response can be directed against a single epitope, a situation that favours selection of mutations allowing escape from immune control 7–9.

*Theileria parva* is one of a number of protozoan parasites, including *Trypanosoma cruzi*, *Toxoplasma gondii* and *Plasmodium* species, for which there is evidence that CD8^+^ T cells are important mediators of immunity 10–13. It is a tick-borne parasite of cattle that infects and transforms lymphocytes causing an acute lymphoproliferative disease, which is a major impediment to livestock production throughout a large part of eastern and southern Africa 14. Since *T. parva* does not infect laboratory animals, generation of information on the mechanisms of immunity has had to rely on studies conducted in the natural host. Treatment of infected animals to ameliorate parasite growth, so-called “infection and treatment”, results in recovery from infection and acquisition of immunity 15. Immunisation of cattle with a single parasite strain provides effective long-lasting immunity against the homologous strain but variable protection against heterologous strains; typically, within a group of immunised animals some are protected whereas others are susceptible to challenge with a heterologous strain 16, 17.

Cattle immunised in this way generate strong CD8^+^ T-cell responses specific for parasitised lymphocytes 18, 19. Experiments involving adoptive transfer of lymphocytes between immune and naïve identical twin calves demonstrated that immunity could be transferred with highly enriched populations of CD8^+^ T cells 12. Further evidence that CD8^+^ T cells are key players in immunity has come from studies of the parasite strain specificity of the responses. CD8^+^ T-cell responses of immune cattle of different MHC genotypes generally display different patterns of parasite strain specificity 20–22. Importantly, the strain specificity of the detectable CD8^+^ T-cell response in animals immunised with one parasite strain has been shown to correlate closely with susceptibility of the animals to subsequent challenge with a second heterologous parasite strain 17.

The genome of *T. parva* is predicted to encode 4034 proteins and analyses of the transcriptome have indicated that over 60% of these proteins are expressed by the intra-lymphocytic schizont stage of the parasite 23, 24. Hence, infected cells contain a large pool of foreign proteins from which, theoretically, many CD8^+^ T-cell epitopes could be generated. In previous studies, we have obtained evidence that MHC-related differences in dominance of target antigens may be an important determinant of the observed variation in the parasite strain specificity of the CD8^+^ T-cell response in *T. parva*-immune animals 25. Recently, several *T. parva* target antigens have been identified by using specific CD8^+^ T-cell lines to screen expressed parasite cDNA 26, 27. A striking feature of the results of these antigen screens was that CD8^+^ T cells from animals of different MHC genotypes tended to identify different parasite antigens.

The present study utilised a subset of these antigens to undertake quantitative analyses of the antigenic specificity of CD8^+^ T-cell responses in cattle immunised by infection and treatment. The results obtained in MHC-homozygous animals demonstrate that a large component of the response is focused on a single dominant antigen, which differs depending on the class I MHC type of the host. Coupled with evidence that the antigens are polymorphic, these findings clearly demonstrate that the observed immunodominance can have a major influence on the parasite strain specificity of the CD8^+^ T-cell response.

## Results

### CD8^+^ T-cell responses of A10^+^ and A18^+^ animals are focused on Tp1 and Tp2

Previous studies have identified several *T. parva* antigens, each of which are recognised by CD8^+^ T cells from immune animals of particular MHC genotypes 26, 27. These include Tp1 and Tp2 presented by class I gene products of the A18 and A10 haplotypes, respectively 28. In order to determine whether responses in A10^+^ and A18^+^ animals also recognise other defined antigens, CD8^+^ T-cell lines from pairs of animals homozygous for A10 and A18 were tested in a cytotoxicity assay against target cells pulsed with pools of overlapping peptides for four or five of the defined *T. parva* antigens. A CD8^+^ T-cell line from an A14-homozygous animal was also included. Cell lines from the A10^+^ and A18^+^ animals exhibited high levels of cytotoxicity against target cells pulsed with Tp2 and Tp1 peptides, respectively, but showed no detectable killing of targets pulsed with the other peptide pools (Table 1). The line from the A14-homozygous animal did not give detectable killing of any of the peptide-pulsed targets. These results demonstrate that responses in A10^+^ and A18^+^ animals are highly focused on Tp1 and Tp2, respectively, in preference to other antigens that generate strong responses in animals of other MHC phenotype.

**Table 1:** Reactivity of CD8^+^ T cell lines, derived from cattle immunised with *T. parva*, with defined overlapping peptide pools representing the Tp1, Tp2, Tp4, Tp5 and Tp8 antigens

Animal	Class I MHC type	Cytotoxicity (%) with peptide-pulsed targetsa)					
		
		Control	Tp1	Tp2	Tp4	Tp5	Tp8
468	A18/A18	0	55	0	0	NT b)	0
641	A18/A18	0	78	0	0	NT b)	0
92	A10/A10	0	0	38	0	0	0
011	A10/A10	0	0	70	0	0	0
605	A14/A14	2	2	3	2	3	2

a) Cytotoxicity of CD8^+^ T cell lines was examined using a 4-hour ^111^In release assay. Target cells consisted of autologous *T. annulata*-infected cells alone (control) or incubated with 1ug/ml of pooled overlapping peptides representing the full-length amino acid sequence of each *T. parva* antigen. Results are shown for assays using an effector to target ratio of 20:1.

b) NT = not tested

### Responding CD8^+^ T cells recognise defined epitopes in Tp1 and Tp2

In previous studies we have identified a single epitope in the Tp1 antigen (Tp1_214–224_) recognised by A18^+^ animals and two epitopes in Tp2 (Tp2_49–59_ and Tp2_98–106_) recognised by A10^+^ animals 28. CD8^+^ T-cell lines from the five MHC-homozygous animals and a further three heterozygous animals, one expressing A10 and two expressing A18, were analysed for reactivity with target cells pulsed with peptides representing these epitopes. The results confirmed previous findings, in that the three A10^+^ animals gave detectable killing of targets pulsed with both Tp2_49–59_ and Tp2_98–106_ and the four A18^+^ animals killed targets pulsed with Tp1_214–224_ (Table 2). The two A10-homozygous animals exhibited stronger responses to Tp2_49–59_ than Tp2_98–106_, whereas the third A10^+^ animal displayed a stronger response to Tp2_98–106_. Again, CD8^+^ T cells from the A14^+^ animal did not kill any of the peptide-pulsed targets.

**Table 2:** Reactivity of CD8^+^ T cell lines, derived from cattle immunised with *T. parva*, with defined epitopes in the Tp1 and Tp2 parasite antigens

Animal	Class I MHC type	Percentage cytotoxicity on target cells a)				
		
		Autologous *T. parva*	Allogeneic *T. parva*	Tp1_214-224_ Peptide	Tp2_49-59_ peptide	Tp2_98-106_ peptide
468	A18/A18	33	1	66	4	3
641	A18/A18	27	4	100	0	0
313	A18/A31	9	1	27	0	0
663	A18/A14	7	2	37	0	0
592	A10/A10	55	3	0	68	15
011	A10/A10	51	2	0	100	47
495	A10/A14	86	1	0	3	30
605	A14/A14	54	4	0	0	0

a) CD8^+^ T cell lines were tested for cytotoxicity using a 4-hour ^111^In release assay. Recognition of peptides was tested using autologous *T. annulata*-infected target cells incubated with 1 ug/mL of peptide. Results are shown for assays using an effector to target ratio of 20:1.

### Tp1 and Tp2 are highly dominant antigens in A18^+^ and A10^+^ animals, respectively

**Figure 1:**
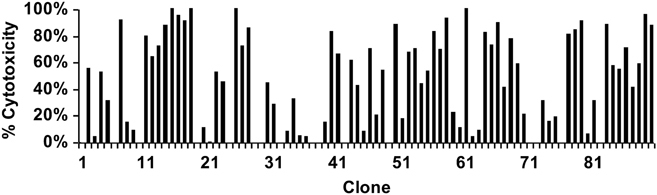
Recognition of the Tp1_214–224_ epitope by CD8^+^ T-cell clones derived from animal 641 (A18/A18): The clones were tested using a 4-h ^111^In-release assay with MHC class I A18^+^, *T. annulata*-infected target cells alone or incubated with 1 μg/mL of Tp1_214–224_ peptide. Results are shown for target cells pulsed with Tp1_214–224_ peptide. The level of killing of unpulsed *T. annulata*-infected target cells was <1% for all clones (data not shown).

**Figure 2:**
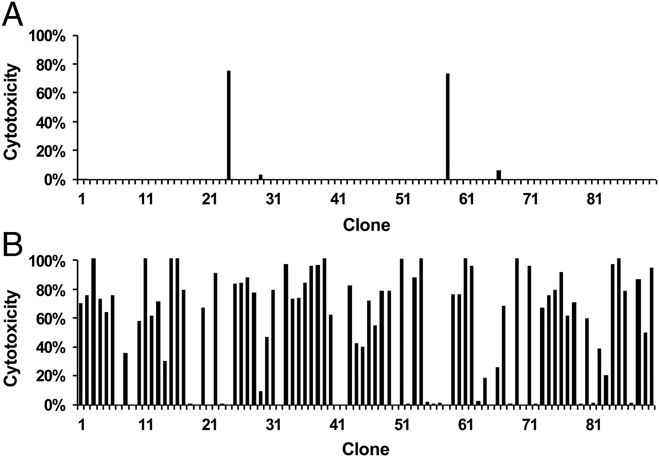
Recognition of the Tp2_49–59_ and Tp2_98–106_ epitopes by CD8^+^ T-cell clones derived from animal 1011 (A10/A10): The clones were tested using a 4-h ^111^In-release assay with MHC class I A10^+^, *T. annulata*-infected target cells incubated with 1 μg/mL of peptide. Results are shown for targets pulsed with Tp2_98–106_ peptide (A) or with Tp2_49–59_ peptide (B).

In order to determine what proportion of the response is directed against the Tp1 and Tp2 antigens, clonal analyses of the CD8^+^ T-cell response were undertaken in two A18-homozygous and two A10-homozygous animals. A panel of 90 T-cell clones from each animal was phenotyped by flow cytometry to exclude contaminant CD3^−^ cells and CD4^+^ and γδ T cells. The CD8^+^ T-cell clones were tested for cytotoxicity on autologous and allogeneic *T. parva*-infected target cells and on target cells pulsed with peptides representing the Tp1 and Tp2 epitopes. Representative results of the analysis of epitope specificity are shown for two animals in Fig. 1 and 2 and a summary of the results for all four animals are presented in Table 3.

**Table 3:** Analyses of CD8^+^ T cell clones derived from animals immunised with *T. parva* for reactivity with defined epitopes in the Tp1 and Tp2 antigens

Animal (MHC type)	Clones tested	Numbers of positive clones and levels of cytotoxicity detected on *T. parva*-infected and peptide-pulsed target cellsa)							
		
		Infected		Tp1_214–224_		Tp2_49–59_		Tp2_98–106_	
					
		Positive	Cytotox	Positive	Cytotox.	Positive	Cytotox.	Positive	Cytotox.
468 (A18/A18)	90	10 (11%)	6–10%	72 (81%)	8–100%				
641 (A18/A18)	87	52 (59%)	6–33%	69 (78%)	7–100%				
592 (A10/A10)	83	53 (64%)	9–74%			49 (59%)	21–96%	3 (4%)	56–99%
1011 A10/A10	89	85 (95%)	7–79%			66 (74%)	9–100%	2 (2%)	73–75%

a) CD8^+^ T cell lines were tested for cytotoxicity using a 4-hour ^111^In release assay. Target cells consisted of autologous *T. parva*-infected cells (infected) and autologous *T. annulata*-infected target cells incubated with 1 ug/mL of the respective peptides. The number and percentage of positive clones (positive) and the range of levels of cytotoxicity of positive clones (cytotox.) are shown for each set of clones on each target cell. A standard cut-off of >5% specific cytotoxicity was used to define positive clones. In all assays, this was well in excess of 3 standard deviations above the mean background release value of the respective target cells and was also well in excess of 3 standard deviations above the mean levels of cytotoxicity obtained with MHC-mismatched *T. parva*-infected targets (as a control for infected targets) and unpulsed *T. annulata*-infected target cells.

A majority of the clones generated from the two A10^+^ animals (64 and 95%) demonstrated detectable MHC-restricted cytotoxicity against the autologous *T. parva*-infected targets, with variable levels of killing ranging from 7 to 79%. Lower levels of specific killing were obtained with clones derived from the two A18^+^ animals (6–33% for 641 and 6–10% for 468) and in animal 468 this was reflected by a much lower proportion of clones (10%) with detectable cytotoxic activity.

**Figure 3:**
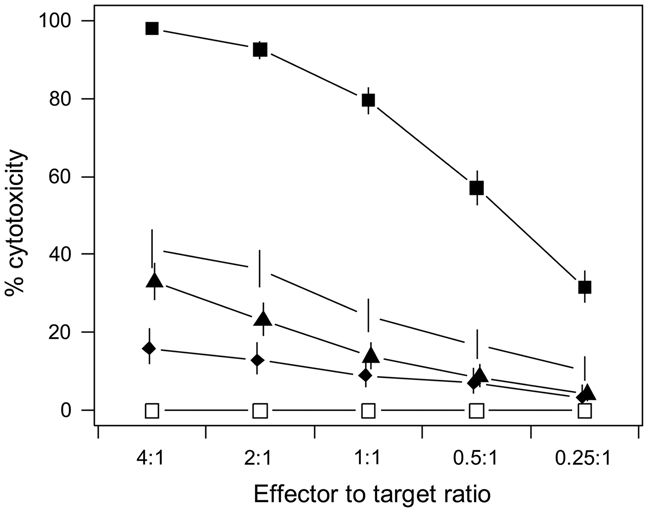
Tp2-specific CD8^+^ T-cell clones show different levels of cytolytic activity for target cells incubated with Tp2_49–59_-peptide: Four T-cell clones (solid symbols) were tested in a 4-h ^111^In-release assay using MHC class I A10^+^, *T. annulata*-infected target cells incubated with 1 μg/mL Tp2_49–59_ peptide. Results obtained using the clones at a range of effector to target ratios are shown (error bars – 95% confidence intervals). Results are also shown for one of the clones tested against the same target cells without added peptide (open squares); similar negative results were obtained with the other clones tested on unpulsed target cells. These assays have been repeated utilising a narrower range of effector to target ratios (2:1 to 0.5:1) and, although the maximal levels of cytotoxicity showed small differences, the relative ranking of levels of cytotoxicity of the clones was similar.

Despite the variable killing of parasitised cells, over 60% of the T-cell clones from all four animals gave significant levels of specific killing of peptide-pulsed targets. In each case, a large majority of the clones was found to be specific for a single epitope. Thus, 78 and 81% of clones from the two A18^+^ animals killed targets pulsed with Tp1_214–224_, and 59 and 74% of clones from the A10^+^ animals reacted with Tp2_49–59_. In the latter two animals, an additional smaller component of the response (4 and 2%) was directed against the second Tp2 epitope (Tp2_98–106_). Additional experiments in which selected clones were tested at a range of effector to target ratios confirmed that the variable levels of killing reflected the cytotoxic activity of the clones rather than variation in the numbers of effectors used in the assays (Fig. 3). In addition, the finding that all clones gave significant killing at an effector to target ratio of 1:1 indicates that the assay employed to screen the epitope specificity of the clones is capable of detecting most clones with cytotoxic activity. These results provide clear evidence that Tp1 and Tp2 are highly dominant antigens in CD8^+^ T-cell responses of A18^+^ and A10^+^ animals to *T. parva*.

### *In vitro* CD8^+^ T-cell lines are representative of the *in vivo* memory population

**Figure 4:**
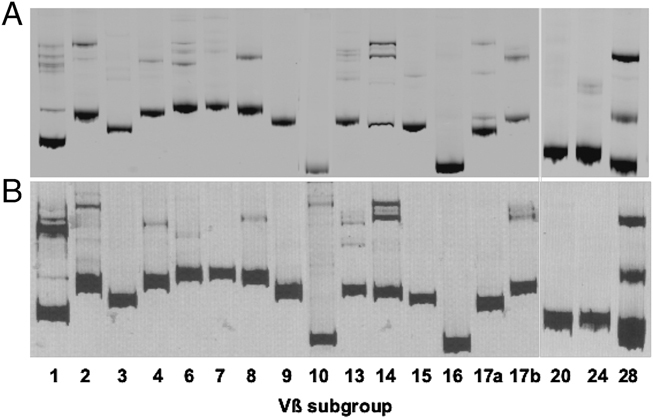
TCR CDR3β heteroduplex analysis of bovine CD8^+^ T-cell responses to *T. parva*: Results obtained for each Vβ subgroup are shown for CD8^+^ T cells harvested *ex vivo* 10 days after parasite challenge of animal 592 (A) and with a CD8^+^ T-cell line derived from the same animal prior to challenge (B). Homoduplexes formed by re-annealing of the carrier DNA strands are represented by a prominent band towards the bottom of each lane. Additional more slowly migrating bands in the upper part of the gel represent heteroduplexes formed from cDNA of expanded clonotypes expressing Vβ genes of the respective subgroup.

**Figure 5:**
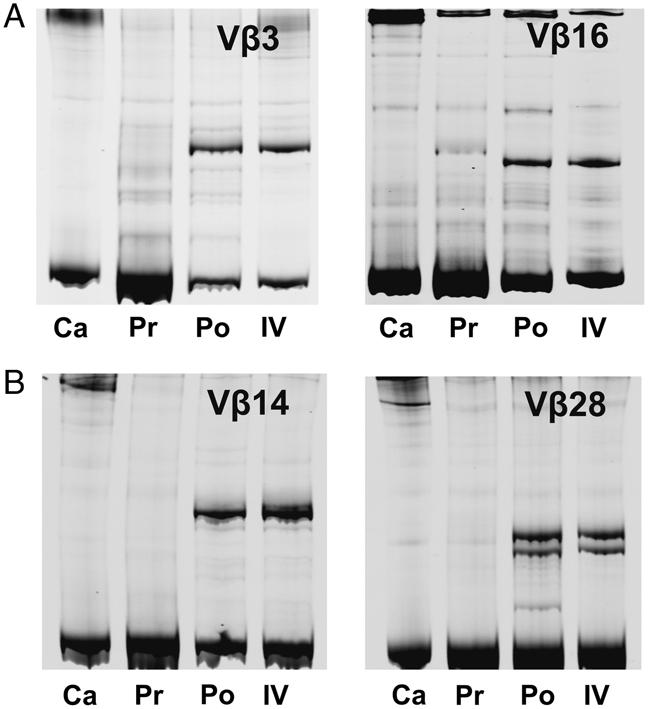
TCR CDR3β heteroduplex analysis of bovine CD8^+^ T-cell responses to *T. parva*: Results are shown for CD8^+^ T cells from animal 641 (A) analysed for subgroups Vβ3 and Vβ16, and for CD8^+^ T cells from animal 011 (B) analysed for subgroups Vβ14 and Vβ28. Lanes labelled Pr represent resting CD8^+^ memory T cells harvested *ex vivo* prior to parasite challenge, those labelled Po represent CD8^+^ T cells harvested *ex vivo* 10 days following challenge and those labelled IV represent a CD8^+^ T-cell line derived immediately prior to challenge. Lanes labelled Ca show the respective heteroduplex carriers loaded with dH20 as a control for bands not related to T-cell populations. Homoduplexes formed by re-annealing of the carrier DNA strands are represented by a prominent band towards the bottom of each lane. Additional more slowly migrating bands in the upper part of the gel represent heteroduplexes formed from cDNA of expanded clonotypes expressing Vβ genes of the respective subgroup.

In order to determine whether these *in vitro* findings reflect the composition of the *in vivo* memory T-cell populations, we used TCR-β chain CDR3 heteroduplex analysis to compare CD8^+^ T-cell lines with CD8^+^ T cells harvested *ex vivo* from the same animals. This assay can distinguish different β chain rearrangements within each Vβ subgroup, based on differential electrophoretic mobility of heteroduplexes formed with a common carrier. Thus, expanded clonotypes within unfractionated T-cell populations yield prominent bands in polyacrylamide gels 29. Three of the MHC-homozygous animals used in this study were challenged on two occasions with a lethal dose of *T. parva* sporozoites. Appropriate samples were not available at the time of the first challenge; therefore, a CD8^+^ T-cell line was derived prior to the second challenge and compared, for each animal, with CD8^+^ T cells harvested *ex vivo* 10 days after challenge, coinciding with the peak of the CD8^+^ T-cell response. Figure 4 shows the results of heteroduplex analysis for all Vβ subgroups for one animal and results for selected Vβ subgroups containing abundant clonal expansions in the other two animals are illustrated in Fig. 5. The assay conditions employed detected only a limited number of faint bands in resting memory CD8^+^ T-cell populations, but detected one or more prominent bands in Vβ subgroups in the CD8^+^ T cells harvested *ex vivo* from actively responding animals. Moreover, in each animal, comparison of the latter with *in vitro*-derived CD8^+^ T cells revealed a remarkably similar Vβ heteroduplex profile. These results demonstrate that the clonal composition of *in vitro* CD8^+^ T-cell cultures is similar to that of the memory population activated *in vivo* following parasite challenge.

### The Tp1 and Tp2 epitopes are polymorphic

**Figure 6:**
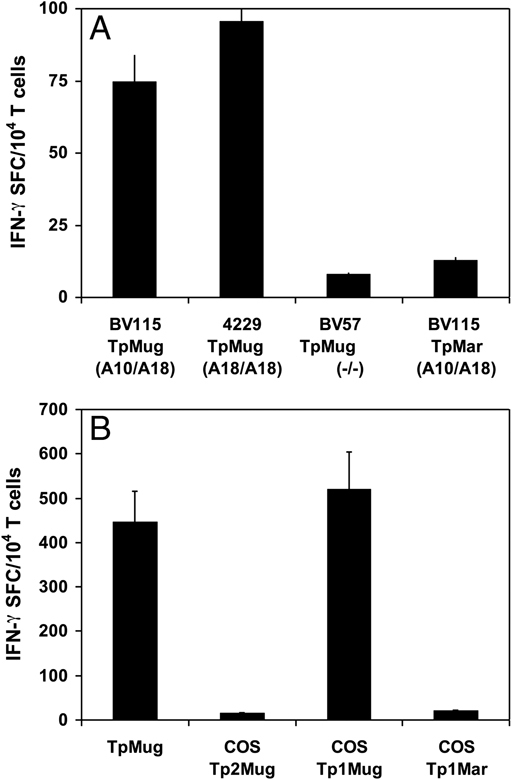
Differential recognition of Tp1 alleles, expressed by the Muguga and Marikebuni isolates of *T. parva*, by a cloned parasite-specific CD8^+^ T-cell line from animal BV115 (A10/A18): (A). A 4-h ^51^Cr-release cytotoxicity assay was used to test reactivity with different parasitised cell lines. Target cells consisted of Muguga-infected cell lines derived from the autologous animal (BV115 Tp Mug), an A18-homozygous animal (4229 Tp Mug) and a class I MHC-mismatched (−/−) animal (BV57 Tp Mug), and a Marikebuni-infected cell line from the autologous animal (BV115 Tp Mar). (B). An IFN-γ ELISpot assay was used to examine recognition of Tp1 cDNA expressed in COS-7 cells. Results (error bars – 95% confidence intervals) are shown for cells co-transfected with the N^*^01301 class I heavy chain together with Muguga Tp2 cDNA (COS Tp2Mug), Muguga Tp1 cDNA (COS TP1Mug) or Marikebuni Tp1 cDNA (COS Tp1Mar). Results obtained using cells infected with *T. parva* (Muguga) (TpMug) as targets are shown for comparison. Data are representative of two independent experiments.

In order to investigate polymorphism of the Tp1 and Tp2 antigens, nucleotide sequences were determined for cloned PCR products of a 435 bp fragment of the Tp1 gene and the full-length Tp2 gene from 27 *T. parva*-infected cell lines, derived from different parasite isolates. Analysis of the Tp1 sequences revealed a sequence from two isolates that differed by a single nucleotide from the Muguga sequence, resulting in one amino acid substitution at position 10 of the Tp1_214–224_ epitope. A further variant sequence obtained from five isolates differed from Muguga by five nucleotides, two of which resulted in amino acid substitutions at positions 10 and 11 in the Tp1_214–224_ epitope (Table 4). The ability of a cloned Tp1-specific CD8^+^ T-cell line to recognise one of the parasitised cell lines that yielded the latter sequence, infected with the Marikebuni isolate, was tested using a cytotoxicity assay. The results, shown in Fig. 6A, demonstrate that this line kills Muguga-infected cells but does not kill Marikebuni-infected cells. Absence of recognition of this Tp1 allele was confirmed by testing the reactivity of the same T-cell line with COS-7 cells co-transfected with the N*01301 class I heavy chain cDNA (which encodes the A18 class I specificity) and either the Muguga or Marikebuni Tp1 genes, using an IFN-γ ELISpot assay. A strong response was observed against Muguga Tp1, but there was no detectable response to Marikebuni Tp1 (Fig. 6B)

**Table 4:** Identification of variation in Tp1 and Tp2 epitopes

Epitope	Parasite	Nucleotide and amino acid sequencesa)										
Tp1_214–224_	Muguga	GTA	GGG	TAT	CCA	AAG	GTT	AAA	GAA	GAA	ATG	CTA
		V	G	Y	P	K	V	K	E	E	M	L
	Kilifi KL1	GTA	GGG	TAT	CCA	AAG	GTT	AAA	GAA	GAA	ATT	CTA
		V	G	Y	P	K	V	K	E	E	I	L
	Marikebuni	GTA	GGG	TAT	CCA	AAG	GTT	AAA	GAA	GAA	ATT	ATA
		V	G	Y	P	K	V	K	E	E	I	I
Tp2_49–59_	Muguga	AAA	TCA	TCA	CAT	GGT	ATG	GGA	AAG	GTA	GGA	AAA
		K	S	S	H	G	M	G	K	V	G	K
	Nyairo BR187	TTA	ACA	TCA	CAT	GGA	ATG	GGA	AAG	ATA	GGT	AGA
		L	T	S	H	G	M	G	K	I	G	R
	Kakuzi 498	TTA	ACA	TCA	CAT	GGT	ATG	GGA	AGG	ATA	GGG	AGA
		L	T	S	H	G	M	G	R	I	G	R
Tp2_98–106_	Muguga	CAA	AGC	CTA	GTG	TGC	GTA	TTA	ATG	AAA		
		Q	S	L	V	C	V	L	M	K		
	Nyairo BR187	GCA	AGC	ATT	AAG	TGT	GTA	TCA	CAC	CAT		
		A	S	I	V	C	V	S	H	H		
	Kakuzi 498	GCA	AGT	ATT	AAG	TGT	GTA	GCA	CAA	TAT		
		A	S	I	K	C	V	A	Q	Y		

a) Substitutions with respect to Muguga are highlighted in grey.

**Figure 7:**
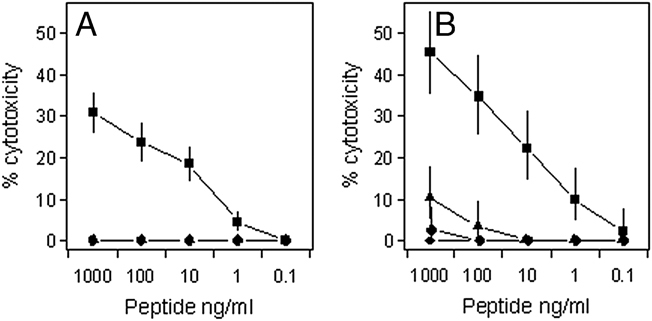
Differential recognition of alleles of the Tp2_49–59_ epitope by two specific CD8^+^ T-cell clones derived from animal 592 (A10/A10): The clones were tested in a 4-h ^111^In-release cytotoxicity assay using autologous *T. annulata*-infected target cells incubated with 1 μg/mL peptide. Results are shown (error bars – 95% confidence intervals) for peptides representing the Muguga Tp2_49–59_ epitope (squares) and the two allelic variants identified in the Nyairo BR187 (diamonds) and Kakuzi 498 (triangles) *T. parva* isolates (see Table 4). Data are representative of two independent experiments. Similar results have also been obtained with additional T-cell clones.

The Tp2 nucleotide sequences showed more extensive sequence divergence. Two pairs of parasite isolates yielded sequences that had identities of only 75.4 and 75.6% with the Muguga genome sequence. Detailed analysis of these sequences will be reported as part of a separate study (R. Pelle *et al*. in preparation). The regions encoding the two epitopes recognised by A10^+^ animals each differed from the Muguga sequence by seven nucleotides in Tp2_49–59_ and 13 or 15 nucleotides in Tp2_98–106_, resulting in four or five amino acid substitutions in Tp2_49–59_ and five or six substitutions in Tp2_98–106_ (Table 4). Recognition of the Tp2_49–59_ variant epitopes by two specific CD8^+^ T-cell clones was examined in a cytotoxicity assay using as target cells a *T. annulata*-infected cell line incubated with synthetic peptides. Both T-cell clones killed target cells pulsed with the native Tp2_49–59_ peptide but did not kill cells pulsed with either of the two variant peptides (Fig. 7). These results show that the Tp1 and Tp2 epitopes are polymorphic and that the allelic variants are differentially recognised by CD8^+^ T cells.

## Discussion

Based on previous observations on strain specificity of CD8^+^ T-cell responses to *T. parva* 17, 21, we have proposed that the response in individual animals is focused on a limited number of the antigens against which the animals are capable of responding. The recent identification of CD8^+^ T-cell target antigens and epitopes has allowed us to address this hypothesis. In the original antigen screens, CD8^+^ T-cell lines from animals of different class I MHC genotypes tended to identify different antigens 26, 27. We had also shown previously that several of the antigens, including Tp1 and Tp2, are recognised consistently by animals of the respective MHC genotypes 28. However, the proportion of the CD8^+^ T-cell response directed towards the respective antigens was not quantified and the possible presence of T cells reactive with the other identified antigens was not examined in detail. The results of the present study demonstrated that responses in MHC-homozygous animals were focused on one of the four or five antigens examined and that >60% of the response was directed to defined epitope(s) in the respective antigens. These findings, coupled with the demonstration that the target epitopes are polymorphic, indicate that such immunodominance can have a major influence on the overall parasite strain specificity of the CD8^+^ T-cell response. Given the evidence that strain specificity of the detectable CD8^+^ T-cell response correlates closely with susceptibility to challenge with heterologous parasite strains 17, these results also imply that the dominant antigens are important targets for protective immunity.

The percentages of T-cell clones found to be specific for the Tp1 and Tp2 epitopes in the present study should be viewed as minimal estimates. Although there was considerable variation between animals in the number of CD8^+^ T-cell clones that gave detectable killing of autologous parasitised cells, a substantial proportion of the “non-cytolytic” clones in some animals gave significant levels of specific killing of peptide-pulsed target cells. The greater sensitivity of the latter target cells probably relates to epitope density, since the concentration of peptides employed is likely to have resulted in a high level of occupancy of class I molecules on the surface of the presenting cells. Heterogeneity in the functional activity within epitope-specific CD8^+^ T-cell populations and in responses to different antigens is a recognised feature of CD8^+^ T-cell responses to viral pathogens 30–33. Hence, it is likely that, in the current study, additional clones specific for the defined epitopes were present among the clones with no detectable cytotoxic activity. Nevertheless, the presence in three of the animals of small subsets of clones that killed parasitised cells but not peptide-pulsed targets indicates that these animals recognise one or more additional undefined antigens.

Since Theileria-infected cell lines express high levels of class I and class II MHC proteins 34 and act as efficient antigen-presenting cells for stimulating parasite-specific memory T cells 20, 35, they are particularly suitable for *in vitro* analyses of T-cell responses to the whole parasite. However, of key relevance to the present study is whether the composition of parasite-specific T-cell lines generated using this *in vitro* system are a true representation of the memory CD8^+^ T-cell population in immunised animals. In order to address this question, we compared the *in vitro* and *in vivo* responses using a TCR-β chain CDR3 heteroduplex assay, which can distinguish clones expressing the same Vβ gene segment but different CDR3 sequences, and thus yields a characteristic molecular profile reflecting the expanded TCR-β chain clonotypes within unfractionated T-cell populations 29. As part of the validation of the assay for analysis of bovine T cells, we had shown that T-cell clones derived from *T. parva*-specific CD8^+^ T-cell lines were broadly representative of the uncloned parent cell line 29. In the present study, comparison of the composition of *ex vivo* CD8^+^ T cells, isolated at the peak of the response to parasite challenge, with CD8^+^ T-cell lines established immediately prior to challenge revealed close similarity in heteroduplex profiles in each animal, indicating that the two populations contain similar repertoires of expanded clonotypes. Of particular relevance was the absence of additional sets of prominent bands in the *in vivo*-derived populations, indicating that the cell lines are representative of the *in vivo* memory CD8^+^ T-cell population activated following parasite challenge.

Despite the antigenic complexity of protozoan parasites, the findings of this study clearly demonstrate that parasite-specific CD8^+^ T-cell responses can display pronounced immunodominance similar to that observed for a number of viral pathogens 5. This represents one of the first documented examples of extreme immunodominance in a natural protozoan disease model. Dominant antigens have been reported recently for CD8^+^ T-cell responses to *T. cruzi* in a mouse model 36, 37. Several epitopes within a large family of *trans*-sialidase (Ts) proteins were consistently recognised by C57BL/6 mice, and a class I tetramer constructed with one of these epitopes was found to stain approximately 30% of CD8^+^ T cells harvested *ex vivo* during the active response to infection. CD8^+^ T cells from *T. cruzi*-infected humans were also found to recognise epitopes in the Ts proteins, although responses in individual patients appeared to be less focused on individual dominant epitopes 36, 38. As with *T. parva* 22, the dominance pattern in mice was found to vary depending on the infective strain of *T. cruzi* 36.

*T. parva* and *T cruzi* both reside free within the cytoplasm of the host cells and hence proteins secreted by, or shed from, the surface of the parasite have direct access to the class I antigen processing pathway. Analysis of the transcriptome of the intra-lymphocytic schizont stage of *T. parva* has detected expression of 2533 (63%) of the 4034 genes predicted from the genome sequence 23, 24 and 405 of the expressed genes (including Tp1 and Tp2) are predicted to encode proteins with a signal peptide or transmembrane domain 24. Evidence from studies in mice indicate that the relative abundance of antigenic proteins and their expression during the early stages of infection may be important factors in determining which proteins are immunodominant 3, 39. In this regard, the *T. cruzi* epitopes that were most dominant in mice were found to be present in multiple members of the Ts protein family 36, 40. Hence, it was proposed that the greater abundance of these epitopes contributed to their dominance. By contrast, the *T. parva* antigens identified to date are all encoded by unrelated single copy genes located in different parts of the genome 26. The evidence that different proteins are dominant in different MHC backgrounds clearly indicates that multiple proteins are available for class I antigen processing and that the dominance is not attributable solely to differences in protein abundance. Studies in murine model systems have implicated a number of other factors, including affinity of peptide-MHC binding, the composition of the TCR repertoire and the affinity of specific TCR for the respective peptide-MHC, in conferring competitive advantage for preferential induction of dominant CD8^+^ T-cell responses (reviewed in 3). In a recent immunisation trial, the observed immunodominance of CD8^+^ T-cell responses to *T. parva* antigens was found to be retained in cattle immunised by a prime-boost protocol with five of the antigens 28. For example, six A18^+^ animals all gave CD8^+^ T-cell responses to Tp1 but had no detectable responses to the other four antigens. Since the antigens were delivered in separate vaccine constructs administered at different sites, it was suggested that the dominance may reflect the presence within the repertoire of T cells with high avidity receptors for the respective epitopes 28.

The practical relevance of immunodominance in CD8^+^ T-cell responses to *T. parva* relates to its potential influence on the parasite strain specificity of the response and cross-protection between parasite strains. Analyses of Tp1 and Tp2 sequences from a limited number of parasite isolates confirmed that these antigens are polymorphic and demonstrated that T-cell clones specific for Muguga Tp1 and Tp2 failed to recognise the respective allelic variant epitopes. Further studies are underway to examine the extent of antigenic diversity in larger samples of parasite isolates and to determine whether the amino acid substitutions affect MHC binding and/or TCR recognition. The induction of highly immunodominant CD8^+^ T-cell responses against one or a few variable antigens, as observed in the present study, clearly has the potential to result in strain-restricted immunity. The observed differences between animals in the patterns of parasite strain specificity of CD8^+^ T-cell responses and immunity are consistent with the finding that responses in animals of different MHC genotypes are focused on different dominant antigens. Such variation in specificity may reflect differences in the extent and nature of polymorphism of different dominant antigens and/or the occurrence of sexual recombination in the parasite population, which would allow independent segregation of alleles of unlinked genes encoding CD8^+^ T-cell antigens. The capacity of *T. parva* to undergo sexual recombination during development in the tick vector has been demonstrated experimentally 41, 42. Moreover, genotyping studies of field isolates of *T. parva* have provided evidence of frequent genetic exchange within the parasite population 43.

The wider applicability of these findings to other related protozoan parasites is unclear. Although several of the CD8^+^ T-cell epitopes in *Plasmodium falciparum* have been shown to be polymorphic 44, their quantitative contribution to parasite-specific CD8^+^ T-cell responses induced by particular parasite strains has been difficult to determine. Partial sequence data from different strains of *T. cruzi* indicate differences in copy number of the dominant Ts epitopes and possible absence of some epitopes in heterologous parasite strains 36. Parasite strain-restricted immunity is well documented for the poultry intestinal protozoan *Eimeria maxima*, and immunodominance of T-cell responses has been proposed to account for the observed variation in strain specificity of immunity in different inbred lines of birds 45. Hence, immunodominance of T-cell responses may be of more general relevance as a determinant of strain restricted immunity in protozoal infections than is currently appreciated.

## Materials and methods

### Animals

Holstein–Friesian cattle carrying different combinations of four defined MHC haplotypes, expressing the A10, A14, A18 and A31 class I serological specificities, were used in this study. The class I types of the animals were determined by a combination of serological typing with class I-specific monoclonal antibodies 46 and class I allele-specific PCR assays 47. They included two pairs of animals, defined as homozygous for the A10 and A18 bovine MHC class I haplotypes. The animals were aged 18–36 months at the outset of the studies. They were reared indoors and maintained on a ration of hay and concentrates.

### Immunisation

Cattle were immunised against the *T. parva* Muguga stock by infection with cryopreserved sporozoites and simultaneous administration of a long-acting formulation of oxytetracycline as described previously 15. Three of the animals were challenged with a lethal dose of sporozoites on two occasions at approximately 18-month intervals following immunisation. The clinical course of infection following immunisation and challenge was followed by monitoring rectal temperature and lymph node enlargement and microscopic examination of smears prepared from needle aspirates of the lymph node draining the site of infection for the presence parasitised cells.

### Parasitised cell lines

Cell lines infected with *T. parva* and *T annulata* were established by infection of PBMC *in vitro* with sporozoites as described previously 48 or by culture of cells from PBMC of infected cattle 49. Cryopreserved sporozoites of the Muguga, Marikebuni, Mariakani, Serengeti and Kiambu 5 isolates of *T. parva* isolates and the C9 cloned population of *T. annulata* were used to establish cell lines. Cell line isolates from naturally infected cattle were obtained from Nyairo in Western Kenya (ten isolates), Kilifi in the Coastal region in Kenya (eight isolates) and Kakuzi in the Central Highlands of Kenya (four isolates). Cloned derivatives of the parasitised cell lines were obtained by limiting dilution as described previously 50.

### Generation of CD8^+^ T-cell lines and clones

*T. parva*-specific CD8^+^ T-cell-enriched cell lines and CD8^+^ T-cell clones were generated as described previously 29. All cultures were conducted in RPMI 1640 medium supplemented with 10% FBS, 20 mM HEPES buffer, 5×10^−5^ 2-mercaptoethanol, 2 mM l-glutamine and 50 μg/mL gentamycin (complete culture medium). Briefly, CD8^+^ T-cell-enriched lines were obtained by stimulation of PBMC twice with gamma-irradiated autologous *T. parva*-infected cells, followed by depletion of CD4 T cells and γδ T cells by antibody and complement-mediated lysis. The remaining cells were stimulated once more with irradiated autologous parasitised cells, in the presence of 100 U/mL recombinant human IL-2 (Chiron, Emeryville, CA., USA), and cloned by limiting dilution 7 days later 50. Clones were expanded by restimulation in 48-well plates to provide sufficient cells to screen different targets for antigenic specificity.

### Cell phenotyping

Cell populations were analysed by single-colour indirect immunoflourescence staining and flow cytometry, using monoclonal antibodies specific for CD3 (MM1A–IgG1), CD4 (IL-A11–IgG2a), CD8 (IL-A51–IgG1) and γδ TCR (GB21A–IgG2b), followed by fluorochrome-labelled mouse isotype-specific secondary reagents (Southern Biotechnology Associates, Birmingham, AL, USA). Stained cells were analysed using a FACScan cell analyser (Becton-Dickinson, Mountain View, CA, USA).

### Cytotoxicity assays

Standard 4-h release assays using ^51^Cr-labelled or ^111^In-labelled target cells were used to measure cytotoxicity of CD8^+^ T-cell lines 50. The antigenic specificity of CD8^+^ T-cell lines was examined using pooled synthetic peptides (16 mer overlapping by 12 residues) covering the full length of five defined *T. parva* antigens (Tp1, Tp2, Tp4, Tp5 and Tp8) and peptides representing three defined peptides in Tp1 and Tp2 (Pepscan Systems, Lelystad, The Netherlands). The latter consisted of an 11-mer epitope in Tp1 (Tp1_214–224_–VGYPKVKEEML) and 11-mer and 9-mer epitopes in Tp2 (Tp2_49–59_–KSSHGMGKVGK; Tp2_98–106_ – QSLVCVLMK) 28. Autologous *T. annulata*-transformed cells were incubated for 60 min with 1 μg/mL of peptide prior to addition of effector cells. All assays were conducted in duplicate and controls included *T. annulata*-infected target cells without added peptide. Because of the large numbers of T-cell clones analysed, the effector T cells used in these assays were not counted, but estimates of cell numbers were made, based on the cell densities in the wells of origin, to ensure that the effector to target cell ratios in the assay were greater than 1:1. In additional experiments, selected clones were tested at defined effector to target ratios.

A standard cut-off of >5% specific cytotoxicity was used to define positive clones. In all assays, this was well in excess of three standard deviations above the mean background release value of the respective target cells and was also well in excess of three standard deviations above the mean levels of cytotoxicity obtained with MHC-mismatched *T. parva*-infected and unpulsed *T. annulata*-infected target cells.

### Analysis of variation in Tp1 and Tp2

Total genomic DNA was prepared from *T. parva*-infected lymphocytes according to Sambrook *et al*. 51. A 435 bp region of the Tp1 open reading frame, containing the A18-restricted epitope, and the full-length coding regions of Tp2 were amplified from genomic DNA by PCR using the following primers: Tp1 forward–ATGGCCACTTCAATTGCATTTGCC; Tp1 reverse–TTCAATGAAATATTTATGAGCTTC; Tp2 forward–ATGAAATTGGCCGCCAGATTA; Tp2 reverse–CTATGAAGTGCCGGAGGCTTC. PCR consisted of 30 ng of genomic DNA and 33 ηg of each primer in a total volume of 33 μL 1× PCR Gold buffer (Applied Biosystems™) containing 1.5 mM MgCl_2_, 200 μM dNTP and 25 U/mL AmpliTaq Gold DNA polymerase (Applied Biosystems™). The cycling conditions were: step 1, 95°C for 11 min; step 2, 95°C for 30 s; step 3, 60°C for 45 s; step 4, 72°C for 30 s (30 times from steps 2 to 4); step 5, 72°C for 10 min. PCR products were sequenced directly (or following cloning into pGEM-T Easy vector (Promega)) on an ABI 3730 capillary sequencer (Applied Biosystems) using specific primers.

Full-length N*01301 MHC class I heavy chain cDNA, amplified from bovine cDNA by PCR using class I-specific forward (ATGRGGCCGCGARCCCW) and reverse (TCAMRCTTTAGGAACYRTGMG) primers, and the Tp1 open reading frame were cloned into the mammalian expression vector pTargeT (Promega). The recombinant plasmids were co-transfected into COS-7 cells and recognition of the expressed products by Tp1-specific CD8^+^ T-cell lines was tested by measuring IFN-γ release using an ELISpot assay as described previously 26.

### Clonal composition of T-cell populations

Cell populations enriched for CD8^+^ T cells were obtained from PBMC by complement-mediated lysis using monoclonal antibodies specific for CD4 and the γδ T-cell marker WC1. The clonal composition of *ex vivo*-derived CD8^+^ T cells and CD8^+^ T-cell lines was examined by analysis of cDNA using a TCR-β chain CDR3 heteroduplex assay as described previously 29. Briefly, transcripts for different bovine Vβ gene subgroups were individually amplified using a set of 18 subgroup-specific 5′ primers along with a conserved 3′ primer in the constant (Cβ) region. Heteroduplex analysis utilised 6-carboxy-fluorescein (FAM)-labelled probes for the respective Vβ subgroups, incorporating the 5′ end (96–279 nucleotides) of the V gene, the CDR3 region and 159 bp of the 3′ end of the constant region. Products were analysed on 9% non-denaturing polyacrylamide gels and results visualised and images stored using a Molecular Imager FX (Biorad, Hercules, CA, USA).

## Abbreviations

Ts: trans-sialidase

## References

[1] Wong P, Pamer EG (2003). CD8 T cell responses to infectious pathogens. Annu. Rev. Immunol..

[2] Mason D (1998). A very high level of cross-reactivity is an essential feature of the T cell receptor. Immunol. Today.

[3] Yewdell JW (2006). Confronting complexity: real-world immunodominance in antiviral CD8^+^ T cell responses. Immunity.

[4] McMichael AJ, Rowland-Jones SL (2001). Cellular immune responses to HIV. Nature.

[5] Yewdell JW, Bennink JR (1999). Immunodominance in major histocompatibility complex class I-restricted T lymphocyte responses. Annu. Rev. Immunol..

[6] Kedl RM, Kappler JW, Marrack P (2003). Epitope dominance, competition and T cell affinity maturation. Curr. Opin. Immunol..

[7] Phillips RE, Rowland-Jones S, Nixon DF, Gotch FM, Edwards JP, Ogunlesi AO, Elvin JG (1991). Human immunodeficiency virus genetic variation that can escape cytotoxic T cell recognition. Nature.

[8] Goulder PJ, Sewell AK, Lalloo DG, Price DA, Whelan JA, Evans J, Taylor GP (1997). Patterns of immunodominance in HIV-1-specific cytotoxic T lymphocyte responses in two human histocompatibility leukocyte antigens (HLA)-identical siblings with HLA-A*0201 are influenced by epitope mutation. J. Exp. Med..

[9] Jones NA, Wei X, Flower DR, Wong M, Michor F, Saag MS, Hahn BH (2004). Determinants of human immunodeficiency virus type I escape from the primary CD8+cytotoxic T lymphocyte response. J. Exp. Med..

[10] Parker SJ, Roberts CW, Alexander J (1991). CD8^+^ T cells are the major lymphocyte subpopulation involved in the protective immune response to *Toxoplasma gondii* in mice. Clin. Exp. Immunol..

[11] Tarleton RI, Koller BH, Latour A, Postan M (1992). Susceptibility of beta-2 microglobulin-deficient mice to *Trypanosoma cruzi* infection. Nature.

[12] McKeever DJ, Taracha ELN, Innes EA, MacHugh ND, Awino E, Goddeeris BM, Morrison WI (1994). Adoptive transfer of immunity to *Theileria parva* in the CD8^+^ fraction of responding efferent lymph. Proc. Natl Acad. Sci. USA.

[13] Marrot A, Zavala F (2004). Effector and memory CD8^+^ T cells as seen in immunity to malaria. Immunol. Rev..

[14] Irvin AD, Morrison WI, Soulsby EJL (1987). Immunopathology, immunology and immunoprophylaxis of Theileria infections. Immune Responses in Parasitic Infections: Immunology, Immunopathology and Immunoprophylaxis.

[15] Radley DE, Brown CGD, Burridge MJ, Cunningham MP, Kirimi IM, Purnell RE, Young AS (1975). East Coast Fever. 1. Chemoprophylactic immunisation of cattle against *Theileria parva* (Muguga) and five *Theileria* strains. Vet. Parasitol..

[16] Radley DE, Brown CGD, Cunningham MP, Kimber CD, Musisi FL, Payne RC, Purnell RE (1975). East Coast fever: 3. Chemoprophylactic immunization of cattle using oxytetracycline and a combination of Theilerial strains. Vet. Parasitol..

[17] Taracha ELN, Goddeeris BM, Morzaria SP, Morrison WI (1995). Parasite strain specificity of precursor cytotoxic T cells in individual animals correlates with cross-protection in cattle challenged with *Theileria parva*. Inf. Immun..

[18] Emery DL, Eugui EM, Nelson RT, Tenywa T (1981). Cell-mediated immune responses to *Theileria parva* (East Coast fever) during immunization and lethal infection in cattle. Immunology.

[19] Morrison WI, Goddeeris BM, Teale AJ, Groocock CM, Kemp SJ, Stagg DA (1987). Cytotoxic T cells elicited in cattle challenged with *Theileria parva* (Muguga): evidence for restriction by class I MHC determinants and parasite strain specificity. Parasite Immunol..

[20] Goddeeris BM, Morrison WI, Teale AJ, Bensaid A, Baldwin CL (1986). Bovine cytotoxic T-cell clones specific for cells infected with the protozoan parasite *Theileria parva*: parasite strain specificity and class I major histocompatibility restriction. Proc. Natl Acad. Sci. USA.

[21] Goddeeris BM, Morrison WI, Toye PG, Bishop R (1990). Strain specificity of bovine *Theileria parva*-specific cytotoxic T cells is determined by the phenotype of the restricting class I MHC. Immunology.

[22] Taracha ELN, Goddeeris BM, Teale AJ, Kemp SJ, Morrison WI (1995). Parasite strain specificity of bovine cytotoxic T cell responses to *Theileria parva* is determined primarily by immunodominance. J. Immunol..

[23] Gardner MJ, Bishop R, Shah T, de Villiers EP, Carlton JM, Hall N, Ren Q (2005). Genome sequence of *Theileria parva*, a bovine pathogen that transforms lymphocytes. Science.

[24] Bishop R, Shah T, Pelle R, Hoyle D, Pearson T, Haines L, Brass A (2005). Analysis of the transcriptome of the protozoan *Theileria parva* using MPSS reveals that the majority of genes are transcriptionally active in the schizont stage. Nucleic Acids Res..

[25] Morrison WI (1996). Influence of host and parasite genotypes on immunological control of *Theileria* parasites. Parasitology.

[26] Graham SP, Pelle R, Honda Y, Mwangi DM, Tonukari NJ, Yamage M, Glew EJ (2006). *Theileria parva* candidate vaccine antigens recognized by immune bovine cytotoxic T lymphocytes. Proc. Natl Acad. Sci. USA.

[27] Graham SP, Honda Y, Pellé R, Mwangi DM, Glew EJ, de Villiers EP, Shah T (2007). A novel strategy for the identification of antigens that are recognised by bovine MHC class I restricted cytotoxic T cells in a protozoan infection using reverse vaccinology. Immunome Res..

[28] Graham SP, Pellé R, Yamage M, Mwangi DM, Honda Y, Mwakubambanya R, de Villiers E (2008). Characterization of the fine specificity of bovine CD8 T cell responses to defined antigens from the protozoan parasite *Theileria parva*. Infect. Immun..

[29] Connelley T, MacHugh ND, Burrells A, Morrison WI (2008). Dissection of the clonal composition of bovine alpha beta T cell responses using T cell receptor Vbeta subfamily-specific PCR and heteroduplex analysis. J. Immunol. Methods.

[30] Veiga-Fernandes H, Walter U, Bourgeois C, McLean A, Rocha B (2000). Response of naïve and memory CD8^+^ T cells to antigen stimulation *in vivo*. Nat. Immunol..

[31] Kaech SM, Wherry EJ, Ahmed R (2002). Effector and memory T-cell differentiation: Implications for vaccine development. Nat. Rev..

[32] Harari A, Dutoit V, Cellerai C, Bart P-A, Du Pasquier RA, Pantaleo G (2006). Functional signatures of protective antiviral T-cell immunity in human virus infections. Immunol. Rev..

[33] Almeida JR, Price DA, Papagno L, Arkoub ZA, Sauce D, Bornstein E, Asher TE (2007). Superior control of HIV-1 replication by CD8+T cells is reflected by their avidity, polyfunctionality and clonal turnover. J. Exp. Med..

[34] De Martini JC, MacHugh ND, Naessens J, Teale AJ (1993). Differential *in vitro* and *in vivo* expression of MHC class II antigens in bovine lymphocytes infected by *Theileria parva*. Vet. Immunol. Immunopathol..

[35] Baldwin CL, Goddeeris BM, Morrison WI (1987). Bovine helper T-cell clones specific for lymphocytes infected with *Theileria parva* (Muguga). Parasite Immunol.

[36] Martin DL, Weatherly DB, Laucella SA, Cabinian MA, Crim MT, Sullivan S, Heiges M (2006). CD8^+^ T cell responses to *Trypanosoma cruzi* are highly focused on strain-variant *trans*-sialadase epitopes. PLoS Pathog..

[37] Tzelepis F, de Alencar BCG, Penido MLO, Claser C, Machado AV, Bruna-Romero O, Gazzinelli RT, Rodrigues MM (2008). Infection with *Trypanosoma cruzi* restricts the repertoire of parasite-specific CD8^+^ T cells leading to immunodominance. J. Immunol..

[38] Alvarez MG, Postan M, Weatherly DB, Albareda MC, Sidney J, Sette A, Olivera C HLA class I T cell epitopes from *trans*-sialidase proteins reveal functionally distinct subsets of CD8 T cells in chronic Chagas disease. PLoS Negl. Trop. Dis..

[39] La Gruta NL, Kedzierska K, Pang K, Webby R, Davenport M, Chen W, Turner SJ, Doherty PC (2006). A virus-specific CD8^+^ T cell immunodominance hierarchy determined by antigen dose and precursor frequencies. Proc. Natl Acad. Sci. USA.

[40] Martin D, Tarleton R (2004). Generation, specificity and function of CD8^+^ T cells in *Trypanosoma cruzi* infection. Immunol. Rev..

[41] Morzaria SP, Young JR, Spooner PR, Dolan TT, Morzaria SP, Bishop RP (1993). *Theileria parva*: a restriction map and genetic recombination. Genomic Analysis of Protozoan Parasites. Morzaria.

[42] Katzer F, Ngugi D, Oura C, Bishop RP, Taracha ELN, Walker AR, McKeever DJ (2006). Extensive genetic diversity in a recombining population of the apicomplexan parasite *Theileria parva*. Infect. Immun.

[43] Oura CAL, Asiimwe BB, Weir W, Lubega GW, Tait A (2005). Population genetic analysis and sub-structuring of *Theileria parva* in Uganda. Mol. Biochem. Parasitol..

[44] Gilbert SC, Plebanski M, Gupta S, Morris J, Cox M, Aidoo M, Kwiatkowski D (1998). Association of malaria parasite population structure, HLA and immunological antagonism. Science.

[45] Smith AL, Hesketh P, Archer A, Shirley MW (2002). Antigenic diversity in *Eimeria maxima* and the influence of host genetics and immunisation schedule on cross-protective immunity. Inf. Immun..

[46] Ellis SA, Morrison WI, MacHugh ND, Birch J, Burrells A, Stear MJ (2005). Serological and molecular diversity in the cattle MHC class I region. Immunogenetics.

[47] Ellis SA, Staines KA, Stear MJ, Henson EJ, Morrison WI (1998). DNA typing for BoLA class I using sequence-specific primers (PCR-SSP). Eur. J. Immunogenet..

[48] Baldwin CL, Black SJ, Brown WC, Conrad PA, Goddeeris BM, Kinuthia SW, Lalor (1988). Bovine T cells, B cells, and null cells are transformed by the protozoan parasite *Theileria parva*. Infect. Immun.

[49] Hulliger L (1965). Cultivation of three species of *Theileria* in lymphoid cells *in vitro*. J. Protozool..

[50] Goddeeris BM, Morrison WI (1988). Techniques for the generation, cloning and characterization of bovine cytotoxic T cells specific for the protozoan *Theileria parva*. J. Tiss. Cult. Methods.

[51] Sambrook J, Fritsch EF, Maniatis T (1989). Molecular Cloning: A Laboratory Manual.

